# Cost-effectiveness of artificial intelligence aided vessel occlusion detection in acute stroke: an early health technology assessment

**DOI:** 10.1186/s13244-021-01077-4

**Published:** 2021-09-25

**Authors:** Kicky G. van Leeuwen, Frederick J. A. Meijer, Steven Schalekamp, Matthieu J. C. M. Rutten, Ewoud J. van Dijk, Bram van Ginneken, Tim M. Govers, Maarten de Rooij

**Affiliations:** 1 Department of Medical Imaging, Radboud University Medical Center, P.O. Box 9101, 6500 HB Nijmegen, The Netherlands; 2 Department of Radiology, Jeroen Bosch Hospital, ‘s-Hertogenbosch, The Netherlands; 3 Department of Neurology, Donders Institute for Brain, Cognition and Behaviour, Centre for Neuroscience, Radboud University Medical Center, Nijmegen, The Netherlands; 4 Department of Operating Rooms, Radboud University Medical Center, Nijmegen, The Netherlands

**Keywords:** Stroke, Artificial intelligence, Cost–benefit analysis, Computed tomography angiography, Endovascular procedures

## Abstract

**Background:**

Limited evidence is available on the clinical impact of artificial intelligence (AI) in radiology. Early health technology assessment (HTA) is a methodology to assess the potential value of an innovation at an early stage. We use early HTA to evaluate the potential value of AI software in radiology. As a use-case, we evaluate the cost-effectiveness of AI software aiding the detection of intracranial large vessel occlusions (LVO) in stroke in comparison to standard care. We used a Markov based model from a societal perspective of the United Kingdom predominantly using stroke registry data complemented with pooled outcome data from large, randomized trials. Different scenarios were explored by varying missed diagnoses of LVOs, AI costs and AI performance. Other input parameters were varied to demonstrate model robustness. Results were reported in expected incremental costs (IC) and effects (IE) expressed in quality adjusted life years (QALYs).

**Results:**

Applying the base case assumptions (6% missed diagnoses of LVOs by clinicians, $40 per AI analysis, 50% reduction of missed LVOs by AI), resulted in cost-savings and incremental QALYs over the projected lifetime (IC: − $156, − 0.23%; IE: + 0.01 QALYs, + 0.07%) per suspected ischemic stroke patient. For each yearly cohort of patients in the UK this translates to a total cost saving of $11 million.

**Conclusions:**

AI tools for LVO detection in emergency care have the potential to improve healthcare outcomes and save costs. We demonstrate how early HTA may be applied for the evaluation of clinically applied AI software for radiology.

**Supplementary Information:**

The online version contains supplementary material available at 10.1186/s13244-021-01077-4.

## Key points

- Early health technology assessment can be used to assess impact of AI.
- The use of AI for large vessel occlusion detection may be cost-effective.
- Increased health and costs savings are expected over the projected lifetime.
- Financial investments and benefits are allocated differently, challenging adoption.

## Background

Currently, there are more than one hundred CE-certified artificial intelligence (AI) software products for radiology on the market, addressing a wide range of applications. Vendors often make broad claims on improving healthcare and increasing efficiency, however evidence on its impact on healthcare is generally lacking [1]. For detection of acute ischemic stroke specifically, six regulatory cleared (CE and/or FDA) AI products are commercially available that detect large vessel occlusions (LVOs) on computed tomography angiography (CTA). The main aim of these products is to improve the diagnostic accuracy of LVOs, while fast and accurate diagnosis is crucial to provide appropriate treatment, especially when intra-arterial thrombectomy (IAT) is possible, which overall results in improved patient outcome [2–4].

There is some evidence on the stand-alone diagnostic accuracy of LVO detection software in peer-reviewed publications (*n* = 2) and FDA submissions (*n* = 3) indicating radiologist-level performance regarding sensitivity [5–9]. However, limited research has yet been published on the added value of these algorithms on the diagnosis, treatment decisions, patient outcomes and societal outcomes [10]. It therefore remains unknown to what extend the use of AI software for LVO detection impacts the diagnostic accuracy and, consequently can impact healthcare outcomes and overall costs.

Early health technology assessment (HTA) is a methodology to assess the potential value of an innovation in an early stage before it has been implemented [11, 12]. The aim of early HTA is to provide insight in the potential value of new technology to inform about further development (is it worthwhile to further develop the technology and perform research on the technology), positioning of the technology within the treatment pathway (for instance should it be an addition to the current pathway or replace something else), required specifications (minimal needed effectiveness, pricing) and future research (what outcomes should be included in future clinical research). Outcomes provide guidance in allocating healthcare resources in an efficient way promoting value-based healthcare. We hypothesize that early HTA is also a useful method to assess the potential impact of AI applications on healthcare outcomes and costs. Therefore, the aim of this study, was to use early HTA to evaluate the potential cost-effectiveness of using an AI tool in ischemic stroke for intracranial LVO detection on computed tomography angiography (CTA) in comparison to standard of care.

## Methods

### Strategies

To demonstrate the potential value of AI-aided LVO detection, two strategies were compared with regards to costs and effects. The first strategy comprised current standard of care. Patients receive head CTA with or without CT perfusion (CTP) when suspected of ischemic stroke based on symptoms and exclusion of other causes as demonstrated by non-contrast CT. The images are evaluated by a radiologist and/or neurologist on duty after which IAT follows if the patient is deemed eligible according to current guidelines [13].

The second strategy was defined as a theoretical strategy in which AI is used as an aid for LVO detection on CTA. For both strategies, only vessel occlusions in the proximal anterior circulation (ICA, A1, M1, M2) were regarded as relevant for patient selection to IAT as in concordance with the recommendations in the current stroke guidelines [13]. In this strategy, we assume that AI software is capable of increasing the diagnostic sensitivity, especially for the detection of M2 occlusions, without a decrease in specificity. False positives of the AI software are expected to be neutralized by the judgement of the reader in order to prevent overtreatment.

Some vendors claim that, besides providing a more accurate diagnosis, the use of AI may lead to shorter time to treatment, especially when it enables to bypass the radiologist [10]. As most currently available commercial products focus on triage and interactive decision support, we only assessed the claim that the use of AI could provide a more accurate diagnosis, i.e. reduce the number of missed LVOs.

### Model structure

**Fig. 1:**
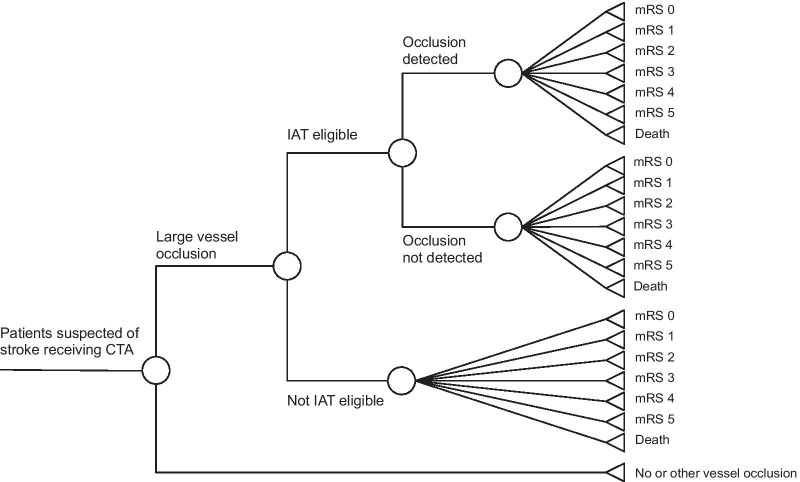
Decision tree applicable to the standard of care strategy and the AI tool strategy. In the AI tool strategy the ratio of occlusions (not) detected was altered. CTA, computed tomography angiography, IAT, intra-arterial thrombectomy

We used a Markov model to demonstrate the costs and health outcomes of the two strategies. The decision tree represents the acute phase (first 90 days) for both strategies (Fig. 1). The modified Rankin Score (mRS), describing the level of disability, at 90 days was the initial state for the subsequent Markov model to model outcomes over a lifetime horizon. After each yearly cycle, over a total of 70 cycles, patients could remain in their current health state, have a recurrent stroke, or die according to mortality probabilities of the general population [14]. The analysis was conducted from a societal perspective in the context of the United Kingdom. Modelling was done in Microsoft Excel 16.

### Population

We based our cohort (*n* = 71,840) predominantly on stroke registry data from the UK [15]. The initial cohort, on which the AI software would be applied, consisted of patients that received CTA in the diagnostic work-up of acute stroke. We excluded late presenters of stroke (last seen well > 4 h) as available mRS data was based on early presenters. Of the patients, 30.6% had a LVO, of whom 43.8% were eligible for IAT treatment [15].

Little is known about the percentage of missed LVOs in standard of care, but estimates ranging between 6 and 20% have been reported in literature [16, 17]. We therefore test a range of this variable in our model and zoom in to the conservative scenario where 6% of LVOs are missed. This number was based on an observer study with three neuroradiologists using CTAs and CTPs for LVO detection [16].

Other variables needed to complete the model were compiled from previously performed large stroke studies. Population age was set at 66 years based on the MR Clean trial [3]. mRS outcomes at 90 days were based on pooled data from large randomized trials (SWIFT PRIME, MR CLEAN, REVASCAT, EXTEND-IA, ESCAPE) [2]. The yearly average probability for recurrent stroke after the acute phase (first 90 days) was 2.84% [18]. Recurrent stroke in the acute phase was included in the mRS outcome at 90 days. In patients who suffered from a recurrent stroke worse outcome with higher mRS states were possible. mRS state distribution in patients with a recurrent stroke were assumed to be equal to a group of patients who did not receive IAT. Table 1 provides an overview of these model inputs.

**Table 1:** Model input—clinical parameters

Model Input	Base case	Reference
LVO missed standard care	6%	Becks et al. [16]
Reduction of missed LVO through AI detection (varied)	50%	
LVO of patients suspected of stroke receiving CTA	30.6%	McMeekin et al. [15]
IAT eligible of LVO population	43.8%	McMeekin et al. [15]
Population age	66	MR Clean Trial [3]
mRS after LVO treated with IAT		Aronsson et al. [2]
mRS 0	11%	
mRS 1	18%	
mRS 2	20%	
mRS 3	17%	
mRS 4	16%	
mRS 5	4%	
Death	14%	
mRS after LVO treated without IAT		Aronsson et al. [2]
mRS 0	5%	
mRS 1	8%	
mRS 2	11%	
mRS 3	17%	
mRS 4	27%	
mRS 5	12%	
Death	20%	
Probability of recurrent stroke	2.84%	Pennlert et al. [18]

LVO, large vessel occlusion; CTA, computed tomography angiography; IAT, intra-arterial thrombectomy; mRS, modified Rankin Scale

### Costs

Costs were derived from a previous study (Table 2) [19]. Short term costs (< 90 days) consisted of costs for treatment, hospitalization and management of adverse events. Long term costs were attributed to the different mRS states, based on the OXVASC study, including costs of personal social services, such as nursing and residential care costs [20].

**Table 2:** Model input—costs

Costs	Base case	Reference
Cost algorithm per analysis (varied)	$40	
Treatment costs*		
Average costs IAT eligible	$11,728	Lobotesis et al. [19], Berkhemer et al. [3], McMeekin et al. [15], Mulder et al. [24]
Average costs non-IAT eligible	$1004	Lobotesis et al. [19], Bandettini di Poggio et al. [25]
Acute stroke costs (< 90 days)		Lobotesis et al. [19]
mRS 0	$4350	
mRS 1	$5117	
mRS 2	$5885	
mRS 3	$22,695	
mRS 4	$30,704	
mRS 5	$36,468	
mRS 6 (death)	$4603	
Long term stroke costs (annually)		Lobotesis et al. [19]
mRS 0	$3936	
mRS 1	$4631	
mRS 2	$5325	
mRS 3	$18,944	
mRS 4	$25,631	
mRS 5	$41,621	
Discount costs	4%	Guideline for economic evaluations in healthcare [21]

^*^Cost of the average treatment was adjusted for the estimated frequency of the treatment according to different studies. For IAT eligible patients, we assumed 85% to receive both IAT and intravenous thrombolysis (IVT), 10% to receive IAT only, and 5% to receive IVT and going for IAT but who appeared revascularised during angiography [3, 15, 24]. For non-IAT-eligible patients we assumed 40% to receive IVT based on previous study and our local medical center data[25]

IAT, intra-arterial thrombectomy; mRS, modified Rankin Scale Costs are rounded to the nearest integer

All costs were inflated to the level of 2019 according to the Consumer Price Index from the Office of National Statistics of the UK and were discounted at 4% annually [21, 22]. We report all costs in US dollars to ease interpretation by applying the 2019 average exchange rate (£1 = $1.283) [23].

### Health outcome

The health outcomes have been described in quality adjusted life years (QALYs) with 0 meaning death and 1 being in perfect health. The mRS scores were utility weighted based on the MR Clean trial (Table 3) [26]. Utilities were discounted at a rate of 1.5% annually [21].

**Table 3:** Model input—utility values

Utility		Dijkland et al. [26]
mRS 0	0.95	
mRS 1	0.93	
mRS 2	0.83	
mRS 3	0.62	
mRS 4	0.42	
mRS 5	0.11	
Discount utilities	1.5%	Guideline for economic evaluations in healthcare[21]

mRS, modified Rankin Scale

### Analysis

In multiple-scenario analyses we assessed the differences in health outcomes and costs at different algorithm performance values (percentage of LVOs detected by the AI tool that would have otherwise been missed), costs of the AI tool (US dollar per analysis) and percentage of missed LVOs in standard of care. A base case analysis was performed using a combination of assumptions for these parameters. The sensitivity of AI tools validated stand-alone for detection of LVOs have been reported to be in the range of 87.8–97.9% [5–8]. However, for this analysis it is relevant to know how much the diagnostic accuracy improves when a radiologist uses the AI tool as a detection aid. As there is no evidence available on the percentage of reduced missed LVOs through the use of commercial AI products, we varied this number around an assumed base case performance of 50%. The price of the AI per case was set at $40 for the base case scenario. As previously described the base case assumption of missed LVOs in standard of care was set at 6%.

The reference value for one QALY was £20,000 ($25,662) [27]. We performed multiple one-way sensitivity analyses in which we varied the parameters that were not included in the scenario analysis to evaluate the robustness of the model.

This was an observational study for which the Institutional Review Board approval was waived. The model is made available on https://www.AIforRadiology.com with the possibility to adapt the variables [28].

## Results

Applying AI for LVO detection has the potential to save costs and increase patient outcomes. Table 4 shows the outcomes in different scenarios regarding the percentage of missed LVOs in usual care and the percentage of missed LVOs that are detected by the innovation. No costs for the innovation were included in these analyses. Therewith, the potential cost savings represent the maximum costs for the innovation at which the innovation is dominant over usual care, i.e., results in both health gain and cost savings. For example, with 1% of current missed LVOs and 25% of detection with the innovation the maximum costs per AI analysis could be $16 for the innovation to be dominant.

**Table 4:** Potential change in costs and QALYs when applying AI tool as opposed to usual care

% LVOs missed usual care	% of missed LVOs detected by innovation				
	1%	25%	50%	75%	100%
1%	− 1$ + 0.00003 QALY	− 16$ + 0.0008 QALY	− 33$ + 0.0016 QALY	− 49$ + 0.0024 QALY	− 65$ + 0.0032 QALY
3%	− 2$ + 0.0001 QALY	− 49$ + 0.0024 QALY	− 98$ + 0.0048 QALY	− 147$ + 0.0071 QALY	− 196$ + 0.0095 QALY
6%	− 4$ + 0.0002 QALY	− 98$ + 0.0048 QALY	− 196$ + 0.0095 QALY	− 294$ + 0.0143 QALY	− 392$ + 0.0190 QALY
10%	− 7$ + 0.0003 QALY	− 163$ + 0.0791 QALY	− 327$ + 0.0158 QALY	− 490$ + 0.0237 QALY	− 654$ + 0.0317 QALY
20%	− 13$ + 0.0006 QALY	− 327$ + 0.0158 QALY	− 654$ + 0.0317 QALY	− 981$ + 0.0475 QALY	− 1,307$ + 0.0633 QALY

Costs and QALYs are per patient receiving CTA with indication stroke, when applying AI tool as opposed to usual care with varying ratio of missed LVOs in current care and varying rates of reduction in missed LVOs due to innovation. No costs for the AI innovation were included in this analysis

QALY, quality-adjusted life-year

Costs are rounded to the nearest integer

**Fig. 2:**
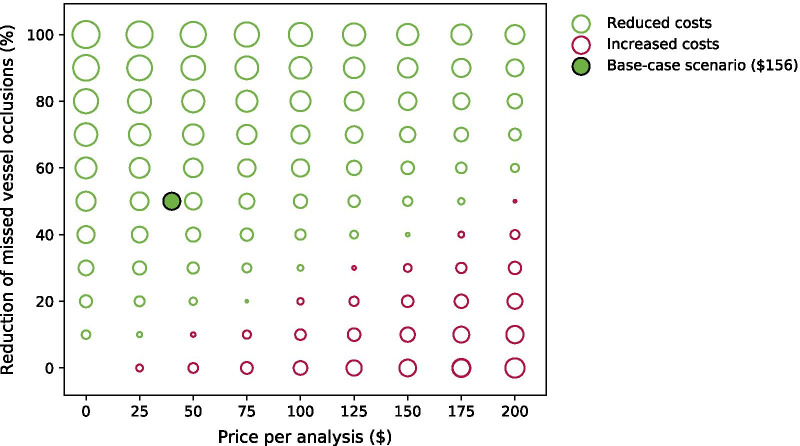
Scenario analysis demonstrating incremental costs. Incremental costs at varying prices for the AI tool per analysis ($0–$200) and varying percentage of reduction of missed large vessel occlusion diagnoses (0–100%). Green circles demonstrate a cost reduction whereas red circles signify an increase in costs. The size of the circle is related to the height of the incremental costs

Figure 2 illustrates the cost saving per patient when varying the costs per AI analysis and the percentage reduction of missed LVOs by the AI tool, assuming a current missed LVO rate of 6%. Here, it becomes apparent at what cost or performance the AI tool will be cost saving. The filled green bullet demonstrates the base case scenario (6% missed diagnoses, $40 per AI analysis, 50% reduction of missed LVOs by AI). Table 5 zooms in on the results of the base case scenario. Here, the model predicted that the AI strategy results in both cost reduction and improved patient outcome compared to the standard of care. For the projected lifetime per ischemic stroke patient, the incremental costs and incremental efficacy were − $156 (− 0.23%) and + 0.0095 QALYs (+ 0.07%) respectively. Using the reference value of $25,662 per QALY, 0.0095 QALY would translate to $244. For each yearly cohort of patients in the UK this translates to a total cost saving of $11 million and QALY gain of 682 ($17.5 million).

**Table 5:** Results of base case analysis* as the difference between usual care and applying the AI tool

	Incremental costs ($)			Incremental efficacy (QALYs)		
	Population	Patient	% of usual care	Population	Patient	% of usual care
Acute phase (< 90 days)	$4,295,152	$60	0.60	9.45	0.0001	0.06
Rest of life phase (> 90 days)	− $15,510,277	− $216	− 0.36	672.82	0.0094	0.07
Total	− $11,215,125	− $156	− 0.23	682.27	0.0095	0.07

^*^Base case parameters: missed LVOs, 6%; costs per analysis, $40; reduction of missed LVOs, 50%

QALY, quality-adjusted life-year

Costs are rounded to the nearest integer

Within a ninety-day window, the intervention scenario led to incremental costs (IC: + $60) due to the increased utilization of the more expensive IAT treatment. Negligible healthcare improvement (IE: + 0.0001 QALY) was observed. A cost reduction and larger QALY gain are expected for the lifetime duration after the acute phase (IC: − $216, IE: + 0.0094 QALY).

**Fig. 3:**
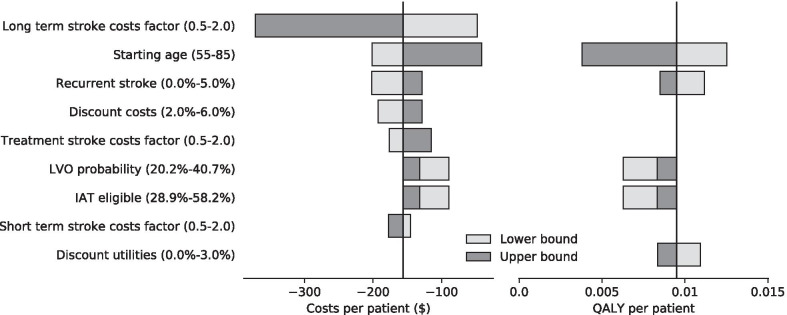
Results of one-way sensitivity analysis. The effect of varying several parameters is shown. In the left diagram the impact on the costs is demonstrated and in the right diagram the impact on the QALYs. Light gray bars represent lower bounds, dark gray bars upper bounds. The axis intersects at the base case results of − $156 and 0.0095 QALY. LVO, large vessel occlusion; IAT, intra-arterial thrombectomy; QALY, quality-adjusted life-year

Figure 3 shows the results of the one-way sensitivity analysis taking the base case scenario as its starting point. In all cases the incremental costs remained negative and the incremental efficacy positive. The long-term stroke costs was the parameter causing most variation in the results followed by the starting age of the patient population. Sensitivity analyses of the mRS probabilities and utility values are reported in the Additional file 1. The analyses did not include extra costs for possible increased reading time for false positive cases as this only resulted in neglectable extra costs − $0.07 for each percentage point of false positives—as shown in the Additional file 2.

## Discussion

We applied early health technology assessment (HTA) to demonstrate the potential cost-effectiveness of an AI tool. Current AI research focuses mainly on the performance of algorithms (the means) instead of assessing the impact on healthcare outcomes and costs, especially in the early stage of development.

In this study, we demonstrate with the use of early HTA that AI software for the aided detection of intracranial LVO in ischemic stroke has the potential to improve healthcare outcomes and save overall costs. The sensitivity analyses demonstrate the robustness to variation of model parameters and assumptions. The improved LVO detection leads to better clinical outcome on both the short term as well as long term resulting in reduced overall healthcare consumption.

Early HTA analyses are not meant to provide a firm ‘go’ or ‘no-go’ recommendation for the development or purchasing of an innovation, but provide insights in the direction to head regarding development, implementation and reimbursement [11]. For example in this study, the analyses show that cost benefits are obtained in the long term, while the costs for the software are short term and are usually covered by the radiology department or hospital. This observation could contribute to the debate on the investments, financial accountability and reimbursement for the clinical use of AI technology.

The difficulty with doing an early HTA is that not all data is yet known, hence it is called ‘early’. Therefore, we used data from previous studies and made some assumptions to complete the model. The sensitivity analysis described, demonstrates the effects when varying the parameters and provides context on the bias and confidence. The model is made publicly available to allow for personalization of the model and the results by adapting the parameter values.

One of the main assumptions was the percentage of missed LVOs. This was assumed to be 6% for the base case scenario, but may vary depending on the clinical context [16, 17]. Because of the limited evidence, we based this rate on a study with two neuroradiologists and one neuroradiology resident as observers. However, in many clinical settings there might not be a specialized physician within reach. Some centers have limited exposure to stroke patients and during out-of-office hours the studies are generally first evaluated by less experienced observers (residents). We therefore consider 6% to be a conservative estimate. With higher percentages of missed LVOs, the benefits of the use of the AI software increase.

Also, we made an assumption on the capability of the AI to reduce missed LVOs in the base case scenario as there is limited evidence of the added value of AI tools in LVO detection. The results of this study only hold, provided that AI tools have a positive impact on the diagnostic accuracy of LVOs. Therefore, we have demonstrated the effects for an AI tool with the capability to reduce the percentage of missed LVOs in the range of 0% to 100%. It should be noted that this percentage cannot be directly translated to the sensitivity of an AI-algorithm applied stand-alone (ranging between 87.8% and 97.9% [5–8]), as we assume that the cases that were missed by a physician are also more likely to be missed by an algorithm (e.g. M2 occlusions). For that reason we went for a conservative 50% reduction of missed diagnoses in the base case scenario and we advise to not use sensitivity measures provided by a vendor directly as the input to this model.

There are some points of note to our analysis. First, in this economic evaluation, we considered a pay-per-use business model and assume costs per analysis. When vendors employ a software license model, the proposed evaluation method can still be applied. By dividing the yearly license fee by the expected population, one obtains an estimate of the per-analysis costs.

Second, we used data from early-presenters as the healthcare outcome data was available for this group only. Currently the indication for IAT is being expanded to also include late presenters [13]. The majority of the UK cohort (86%) was made up of early presenters. We hypothesize that cost savings and health gain remain positive, but will be slightly reduced when including the late presenters in the model. Other AI tools aimed at assessing eligibility for IAT, such as CTP analysis, collateral status assessment, or ASPECT scoring may have more impact on the late presenting population, but are beyond the scope of this study.

Lastly, we did not elaborately take into account the interaction between the physician and the AI software. We expect limited effects of false positives when present at an acceptable rate. We assumed that false positive predictions of the AI software would be neutralized by the reader and would not lead to unnecessary invasive procedures (diagnostic subtraction angiogram). False positives may contribute to a slightly longer reading time, while true positives may speed up the reading process. As shown in the sensitivity analyses the slightly extra reading time resulted in neglectable extra costs.

In this work, we compared standard of care with use of an AI tool to increase diagnostic efficacy in LVO detection in ischemic stroke. Depending on the center and the way that stroke care is organized, there may be alternative strategies or a combination of strategies to reach the same goal that we did not consider, such as the use of advanced imaging techniques including CTP, additional training of the physicians interpreting the CTA scans, a dedicated (neuro)radiologist on call outside of office hours, or even other types of AI tools. In further cost-effectiveness studies these alternatives could be explored to determine if implementing an AI tool for LVO detection is the most cost-efficient solution.

## Conclusion

We demonstrate the potential cost-effectiveness of computer aided LVO detection in ischemic stroke by performing an early health technology assessment. Such analysis can be used to indicate the potential efficacy of an AI tool in an early phase to guide development and implementation. The most important next step is to validate the outcomes of the early health technology assessment in clinical practice. With increasing number of AI tools implemented in the clinic, it is important to assess the impact of AI tools on our healthcare system. Real-life outcome measures should be used to gain insights into how to apply AI tools in a sensible and safe way. This is a prerequisite to prove the claim that AI is making healthcare better and more affordable.

## Supplementary Information

**Additional file 1.** Sensitivity analysis of mRS distributions and utility values.

**Additional file 2.** Costs false positives.

## Data Availability

The model is made available on on https://www.AIforRadiology.com with the possibility to adapt the variables.

## Abbreviations

AI: Artificial intelligence
CTA: Computed tomography angiography
CTP: Computed tomography perfusion
HTA: Health technology assessment
IAT: Intra-arterial thrombectomy
IC: Incremental costs
IE: Incremental effects
IVT: Intravenous thrombolysis
LVO: Large vessel occlusion
QALY: Quality adjusted life year

## References

[1] van Leeuwen KG, Schalekamp S, Rutten MJCM, van Ginneken B, de Rooij M (2021). Artificial intelligence in Radiology: 100 commercially available products and their scientific evidence. Eur Radiol.

[2] Aronsson M, Persson J, Blomstrand C, Wester P, Levin L-Å (2016). Cost-effectiveness of endovascular thrombectomy in patients with acute ischemic stroke. Neurology.

[3] Berkhemer OA, Fransen PSS, Beumer D (2015). A randomized trial of intraarterial treatment for acute ischemic stroke. N Engl J Med.

[4] Goyal M, Menon BK, van Zwam WH (2016). Endovascular thrombectomy after large-vessel ischaemic stroke: a meta-analysis of individual patient data from five randomised trials. Lancet.

[5] (2019) Aidoc Medical, Ltd.- BriefCase LVO - 510(k). U.S. Food & Drug Administration

[6] (2018) Viz.Al, Inc. - ContaCT - De Novo. U.S. Food & Drug Administration

[7] (2020) Avicenna.ai - CINA - 510(k). U.S. Food & Drug Administration

[8] Amukotuwa SA, Straka M, Smith H (2019). Automated detection of intracranial large vessel occlusions on computed tomography angiography. Stroke.

[9] Amukotuwa SA, Straka M, Dehkharghani S, Bammer R (2019). Fast automatic detection of large vessel occlusions on CT angiography. Stroke.

[10] Hassan AE, Ringheanu VM, Rabah RR, Preston L, Tekle WG, Qureshi AI (2020). Early experience utilizing artificial intelligence shows significant reduction in transfer times and length of stay in a hub and spoke model. Interv Neuroradiol.

[11] Grutters JPC, Govers T, Nijboer J, Tummers M, van der Wilt GJ, Rovers MM (2019) Problems and promises of health technologies: the role of early health economic modeling. Int J Health Policy Manag 8:575–582. 10.15171/ijhpm.2019.36 10.15171/ijhpm.2019.36 PMC6819627 31657184

[12] Ijzerman MJ, Steuten LMG (2011). Early assessment of medical technologies to inform product development and market access. Appl Health Econ Health Policy.

[13] Powers WJ, Rabinstein AA, Ackerson T (2019). Guidelines for the Early Management of Patients With Acute Ischemic Stroke: 2019 Update to the 2018 Guidelines for the Early Management of Acute Ischemic Stroke: A Guideline for Healthcare Professionals From the American Heart Association/American Stroke Association. Stroke.

[14] (2017) Mortality probabilities. Statistics Netherlands’ database (CBS)

[15] McMeekin P, White P, James MA, Price CI, Flynn D, Ford GA (2017). Estimating the number of UK stroke patients eligible for endovascular thrombectomy. Eur Stroke J.

[16] Becks MJ, Manniesing R, Vister J (2019). Brain CT perfusion improves intracranial vessel occlusion detection on CT angiography. AJNR Am J Neuroradiol.

[17] Fasen BACM, Heijboer RJJ, Hulsmans F-JH, Kwee RM (2020). CT angiography in evaluating large-vessel occlusion in acute anterior circulation ischemic stroke: factors associated with diagnostic error in clinical practice. AJNR Am J Neuroradiol.

[18] Pennlert J, Eriksson M, Carlberg B, Wiklund PG (2014). Long-term risk and predictors of recurrent stroke beyond the acute phase. Stroke.

[19] Lobotesis K, Veltkamp R, Carpenter IH, Claxton LM, Saver JL, Hodgson R (2016). Cost-effectiveness of stent-retriever thrombectomy in combination with IV t-PA compared with IV t-PA alone for acute ischemic stroke in the UK. J Med Econ.

[20] Luengo-Fernandez R, Yiin GSC, Gray AM, Rothwell PM (2013). Population-based study of acute- and long-term care costs after stroke in patients with AF. Int J Stroke.

[21] (2016) Guideline for economic evaluations in healthcare. National Health Care Institute, The Netherlands *

[22] (2019) Consumer Price Index UK. Office of National Statistics of the UK

[23] (2019) British Pound (GBP) to US Dollar (USD) exchange rate history

[24] Mulder MJHL, Jansen IGH, Goldhoorn R-JB (2018). Time to endovascular treatment and outcome in acute ischemic stroke. Circulation.

[25] Bandettini-di-Poggio M, Finocchi C, Brizzo F (2019). Management of acute ischemic stroke, thrombolysis rate, and predictors of clinical outcome. Neurol Sci.

[26] Dijkland SA, Voormolen DC, Venema E (2018). Utility-weighted modified rankin scale as primary outcome in stroke trials. Stroke.

[27] (2012) Methods for the development of NICE public health guidance (third edition). National Institute for Health and Clinical Excellence, United Kingdom 27905711

[28] Diagnostic Imaging Analysis Group (2020) AI for Radiology. Radboud university medical center. https://www.AIforRadiology.com. Accessed 15 Jan 2021

