# Supplementary material for: Cost-effectiveness of artificial intelligence aided vessel occlusion detection in acute stroke: an early health technology assessment
Source: Insights Imaging. 2021 Sep 25;12:133. doi: 10.1186/s13244-021-01077-4 (PMC8464539; doi:10.1186/s13244-021-01077-4)
Supplement: Supplementary file 1 — Additional file 1. Sensitivity analysis of mRS distributions and utility values. [file 13244_2021_1077_MOESM1_ESM.docx]

**ELECTRONIC SUPPLEMENTARY MATERIAL**

**Cost-effectiveness of Artificial Intelligence Aided Vessel Occlusion Detection in Acute Stroke: an Early Health Technology Assessment**

## Sensitivity analysis of mRS distributions and utility values

We analyzed the robustness of the model to uncertainty of the mRS probability distributions and utility values by skewing the distributions (table I). In scenario a and c the lower mRS score probabilities were multiplied by a factor of 0.7. The probabilities with the higher mRS scores were proportionally increased to get to a total of 100%. A similar approach was take for scenario b and d, however, with a factor of 1.3 skewing the distribution to the lower mRS scores. For scenario e and f the mean between two subsequent utility values was taken to skew the distributions in both directions. The effect of the different scenarios on cost and healthcare outcomes is demonstrated in figure I.


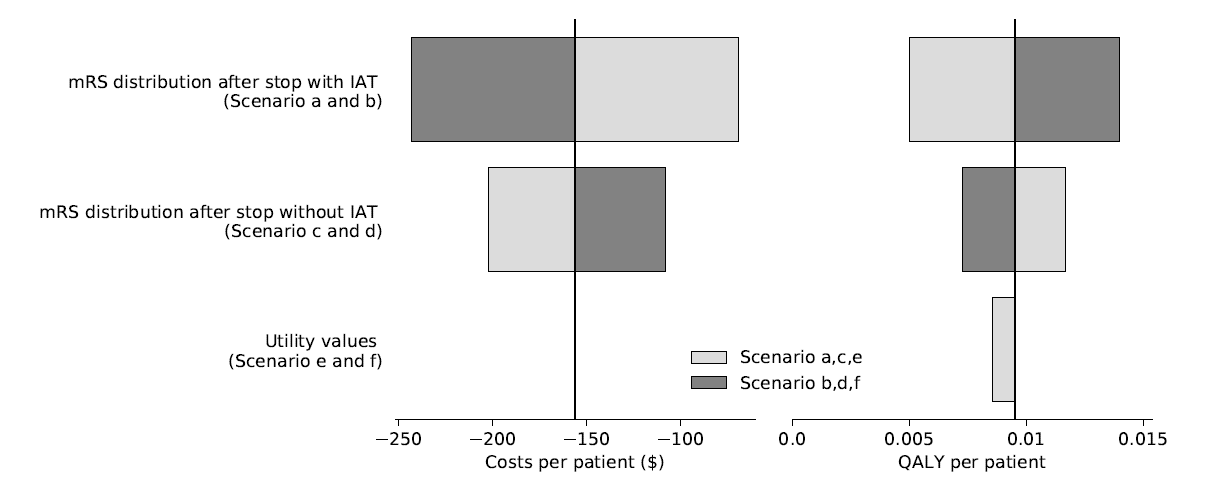


**Figure I. *Sensitivity analysis of varying mRS probabilities and utility values****. The effect of the different scenarios described in table S1 on costs and health outcomes. In the left diagram the impact on the costs is demonstrated and in the right diagram the impact on the QALYs. Light grey bars represent scenario a, c and e, dark grey bars b, d and f as described in table S1. The axis intersects at the base case results of -$156 and 0.0095 QALY.*

*mRS = modified Rankin Scale, IAT=intra-arterial thrombectomy, QALY=quality-adjusted life-year*

**Table I. *Skewed distributions for mRS probabilities and utility values***

| **Distribution after stop with IAT** |  |  |  |
| --- | --- | --- | --- |
|  |  | **Scenario a** | **Scenario b** |
|  | **base case** | **mRS 0, 1, 2 < (x0**.**7)** | **mRS 0, 1, 2 > (x1**.**3)** |
| mRS 0 | 11.0% | 7.7% | 14.3% |
| mRS 1 | 18.0% | 12.6% | 23.4% |
| mRS 2 | 20.0% | 14.0% | 26.0% |
| mRS 3 | 17.0% | 21.9% | 12.1% |
| mRS 4 | 16.0% | 20.6% | 11.4% |
| mRS 5 | 4.0% | 5.2% | 2.8% |
| dead | 14.0% | 18.0% | 10.0% |
| **Distribution after stop without IAT** |  |  |  |
|  |  | **Scenario c** | **Scenario d** |
|  | **base case** | **mRS 0, 1, 2 < (x0**.**7)** | **mRS 0, 1, 2 > (x1**.**3)** |
| mRS 0 | 5.0% | 3.5% | 6.5% |
| mRS 1 | 8.0% | 5.6% | 10.4% |
| mRS 2 | 11.0% | 7.7% | 14.3% |
| mRS 3 | 17.0% | 18.6% | 15.4% |
| mRS 4 | 27.0% | 29.6% | 24.4% |
| mRS 5 | 12.0% | 13.1% | 10.9% |
| dead | 20.0% | 21.9% | 18.1% |
| **Quality of life (utility)** |  | **Scenario e** | **Scenario f** |
|  | **base case** | **skew utility to low mRS** | **skew utility to high mRS** |
| mRS 0 | 0.950 | 0.950 | 0.940 |
| mRS 1 | 0.930 | 0.940 | 0.880 |
| mRS 2 | 0.830 | 0.880 | 0.725 |
| mRS 3 | 0.620 | 0.725 | 0.520 |
| mRS 4 | 0.420 | 0.520 | 0.265 |
| mRS 5 | 0.110 | 0.265 | 0.110 |

*mRS = modified Rankin Scale*

$$Costs per false positive=\frac{hourly rate}{60}\times extra time needed= \frac{\$122}{60 min}\times5 min=\$10.16$$

$$Costs per patient=costs per false positive\times negative rate\times false positive rate=\$10.16\times69.4\%\times1\%=\$0.07$$
