# Supplementary material for: Cost-effectiveness of artificial intelligence aided vessel occlusion detection in acute stroke: an early health technology assessment
Source: Insights Imaging. 2021 Sep 25;12:133. doi: 10.1186/s13244-021-01077-4 (PMC8464539; doi:10.1186/s13244-021-01077-4)
Supplement: Supplementary file 2 — Additional file 2. Costs false positives. [file 13244_2021_1077_MOESM2_ESM.docx]

**ELECTRONIC SUPPLEMENTARY MATERIAL**

**Cost-effectiveness of Artificial Intelligence Aided Vessel Occlusion Detection in Acute Stroke: an Early Health Technology Assessment**


## Costs false positives

False positives may increase reading time by the radiologist. In case each false positive may take an extra 5 minutes by a radiologist to assess the scan, with an hourly rate of 122 dollar the cost per false positive is $10.16.

$$Costs per false positive=\frac{hourly rate}{60}\times extra time needed= \frac{\$122}{60 min}\times5 min=\$10.16$$

Per patient within the population this would result in $0.07 of extra costs, for each percent of false positives, with a negative rate of 69.4%.

$$Costs per patient=costs per false positive\times negative rate\times false positive rate=\$10.16\times69.4\%\times1\%=\$0.07$$

At a specificity of 90% and thus a 10% false positive rate this would result in $0.70 per patient. This is neglectable in the context of the base case scenario in which total cost savings are predicted to be $156 per patient.
